# Development of Vaginal Carriers Based on Chitosan-Grafted-PNIPAAm for Progesterone Administration

**DOI:** 10.3390/gels8090596

**Published:** 2022-09-17

**Authors:** Oana-Teodora Afloarea, Catalina Natalia Cheaburu Yilmaz, Liliana Verestiuc, Nela Bibire

**Affiliations:** 1 Department of Analytical Chemistry, Faculty of Pharmacy, “Grigore T. Popa” University of Medicine and Pharmacy, 700115 Iaşi, Romania; 2 “Petru Poni” Institute of Macromolecular Chemistry, 41 A Grigore Ghica Voda Alley, 700487 Iaşi, Romania; 3 Department of Biomedical Sciences, Faculty of Medical Bioengineering, “Grigore T. Popa” University of Medicine and Pharmacy, 700115 Iaşi, Romania

**Keywords:** vaginal, progesterone, miscarriage, hydrogel, chitosan, graft copolymer, PNIPAAm, RAFT polymerization, freeze-thaw

## Abstract

Chitosan-based hydrogels possess numerous advantages, such as biocompatibility and non-toxicity, and it is considered a proper material to be used in biomedical and pharmaceutical applications. Vaginal administration of progesterone represents a viable alternative for maintaining pregnancy and reducing the risk of miscarriage and in supporting the corpus luteum during fertilization cycles. This study aimed to develop new formulations for vaginal administration of progesterone (PGT). A previously synthesized responsive chitosan-grafted-poly (N-isopropylacrylamide) (CS-g-PNIPAAm) was formulated in various compositions with polyvinyl alcohol (PVA) as external crosslinking agent to obtain pH- and temperature-dependent hydrogels; the hydrogels had the capacity to withstand shear forces encountered in the vagina due to its mechanism of swelling once in contact with vaginal fluids. Three different hydrogels based on grafted chitosan were analyzed via Fourier-transform infrared spectroscopy (FTIR), swelling tests, in vitro drug release, and bioadhesion properties by TA.XTplus texture analysis. A higher amount of PVA decreased the swelling and the bioadhesion capacities of the hydrogel. All hydrogels showed sensitivity to temperature and pH in terms of swelling and in vitro delivery characteristics. By loading progesterone, the studied hydrogels seemed to possess even higher sensitivity than drug–free matrices. The release profile of the active substance and the bioadhesion characteristics recommended the CS-g-PNIPAAm/PVA 80/20 +PGT (P1) hydrogel as a proper constituent for the vaginal formulation for progesterone administration.

## 1. Introduction

Hydrogels are a category of smart biomaterials with remarkable therapeutic applications. The potential of hydrogel application for various therapies using especially natural polymers is being widely explored to obtain an effective strategy to deliver the active substance (drug) at a constant rate to the site of action and to achieve optimal bioavailability of the therapeutic agent. These controlled release systems have a three-dimensional structure which is made of reticular polymers that enhance performance (degree of swelling, bioadhesion, etc.) while ensuring biofunctionality and biosafety (no cytotoxicity, mutagenesis, or other toxic effect) [1,2,3] together with a high potential to absorb water or biological fluids being called as superabsorbent hydrogels [4].

Due to their provenance, natural polymers possess low solubility in water and solvents, weak mechanical characteristics and thermal stability; these drawbacks may be adjusted by their chemical modification without affecting their biocompatible and non-toxic character. More particularly, even after cross-linking processes, natural based polymers forming hydrogels can still be considered compatible and harmless, providing bifunctionality and biosafety for the host (does not cause cytotoxicity, mutagenesis, or other harmful effects) [5].

A key feature of hydrogels is mucoadhesion, a phenomenon which involves two phases, a contact phase and a consolidation phase, following the interaction between the muco-adhesive polymer and the mucosa [6]. The mucoadhesion process has many advantages: prolonged contact time enhances absorption, leading to an increased therapeutic efficacy; a large amount of blood and good blood flow rate causes rapid drug absorption; avoidance of drug degradation due to the acidic environment in the gastro-intestinal tract; avoidance of hepatic metabolism; and faster onset of action due to the mucosal surface [7].

Nowadays, due to various factors (e.g., environmental, genetic, diet) humans are facing new challenges related to the reproduction ability and maintaining to the term of healthy pregnancy. Vaginal administration of drugs is a viable alternative for maintaining pregnancy and reducing the risk of miscarriage. Progesterone therapy plays a key role in preventing preterm birth in pregnancies with a shortened cervix, as well as in supporting the corpus luteum during fertilization cycles [8]. Progesterone is produced by the cells of the ovarian corpus luteum, and during pregnancy by the placenta [9]. Currently, vaginal administration of progesterone is considered a better option because it has a direct effect on the vagina-uterus area, also known as “first uterine pass effect”. It has been observed that oral administration of micronized progesterone has a reduced bioavailability <10%, because before entering the systemic route the permeability of the drug is affected by the gut microbiome and the hepatic first pass effect [10,11]. Micronized progesterone therapy on each bone requires an additional dose to maintain a serum level of protection against the endometrium, as the pharmacokinetic performance decreases especially during pregnancy, resulting in a decrease in the peak plasma concentration as well as a reduction in the time at which the peak plasma concentration occurs [12]. Side effects of oral progesterone include drowsiness, headache, abdominal cramps, constipation, breast tenderness, nausea, dizziness, oedema, hypotension, dysphoria, and vaginal dryness [13,14]. According to clinical studies, vaginal administration has an optimal therapeutic effect and may reduce side effects in women at risk of preterm birth. Due to its anti-inflammatory effect, progesterone can reduce uterine contractility and in the case of in vitro fertilization it supports the luteal phase [13]. The risk of preterm birth is significantly decreased for pregnant women at 28–34 weeks. Neonatal outcomes showed a decrease in perinatal death, higher birth weight, and shorter length of stay in intensive care, with no adverse effects on neurodevelopment. Therefore, progesterone has the potential to have a beneficial systemic effect compared to placebo [15,16]. In the recent years, there are various strategies to develop hydrogels for localized therapy. The prepared hydrogel is based on a natural polymer: chitosan, due to its mimetic properties with the extracellular biological matrix which provides better tolerability and safety of the biological product used. Chitosan is recognized as a safe substance (GRAS) by the Food and Drug Administration (FDA) Chitosan contains a cationic amino group, and this unique characteristic allows it to be easily complexed with anionic molecules to form hydrogels. Modification of the hydroxyl and amino group has been sought to improve the physicochemical and biological properties of chitosan [17,18,19]. The structure of chitosan influences its interaction with mucin from cervicovaginal mucus. Cervicovaginal mucus has a typical acidic pH (3.5–4.5) and the main components of secreted mucus are soluble mucins. These are highly glycosylated glycoproteins consisting of approximately 500 kDa units, which subunify via disulfide bridges to give large water-wrapping structures, with the role of providing a viscoelastic protective layer on the mucosal surface [20].

**Figure 1:**
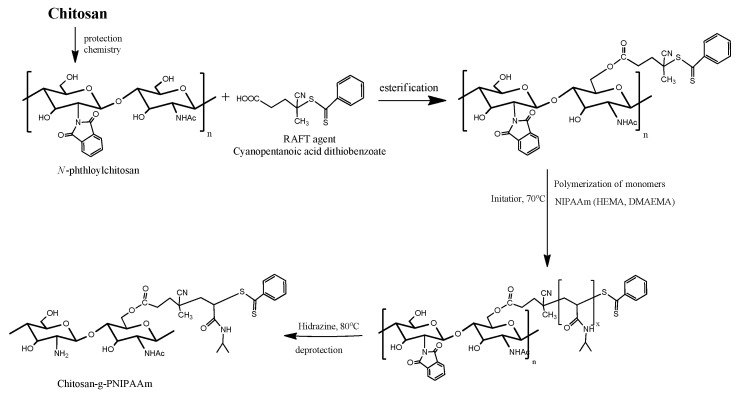
Synthesis approach of the CS-g-PNIPAAm via RAFT polymerization.

The use of polyvinyl alcohol together with chitosan aimed to increase the degree of adhesion by interpenetrating hydroxyl groups with amino groups [21]. Physically, cross-linked PVA hydrogels are the most widely used in the biomedical field due to its high level of purity, biocompatibility, mechanical strength, and especially by its simple gelling mode; PVA solutions turn into gels upon repetitive freeze-thaw cycles because of phase separation and crystallization [22]. By incorporating synthetic polymers within the natural polymer’s chains, particularly with stimuli responsive characteristics, it may lead to a more complex architecture with improved properties such as mucoadhesivity and controlled release capacity. Temperature-sensitive hydrogels are the most widely used due to their high potential for application in controlled drug release systems. Poly(N-isopropylacrylamide) (PNIPAAm) is a homopolymer network obtained by controlled polymerization of N-isopropylacrylamide (NIPAAm) monomers [23,24]. Graft copolymers of chitosan and poly(N-isopropylacrylamide) (PNIPAAm) showed previously that it may form a viscous hydrogel adherent to the contact surface [19]. Figure 1 describes the proposed mechanism of the chemical modification of chitosan [25].

The interaction between CS-graft-poly(N-isopropylacrylamide) modified chitosan and polyvinyl alcohol (PVA) was studied previously by our research group [19] and it resulted in a pH- and temperature-dependent hydrogel for antifungal treatment in topical semisolid formulations. Additionally, similar systems showed good capacity to resist the shear forces encountered due to its mechanism of swelling once in contact with body fluids from mucosal substrate. After this initial “contact” stage, the hydrogel can “strengthen” its adhesion to the mucosa with additional physical and chemical interactions [19,21,26,27].

The present study aimed at the preparation and characterization of formulations as hydrogels containing chemically modified chitosan (CS-graft-poly(N-isopropylacrylamide)), PVA within various ratios and progesterone, as drug model for vaginal administration’s purpose. We planned to design a therapeutic alternative for intravaginal administration of progesterone, targeting the therapy of pregnant women at high risk of miscarriage and those performing in vitro fertilization. The idea of the study came from preliminary studies already performed for oral mucosa and, as the non-toxicity of the polymeric carrier was proven, the same type of system could be applied for intravaginal treatment and avoiding unnecessary additional animal testing. Regarding the novelty of the study, still a controlled and scalable method for chitosan modification was not further developed; the development of these systems represents an advantage regarding the practical application, as the same polymeric vehicle could be used successfully for different types of formulations and on different substrates with similar results.

## 2. Results and Discussion

### 2.1. FTIR Spectroscopy

**Figure 2:**
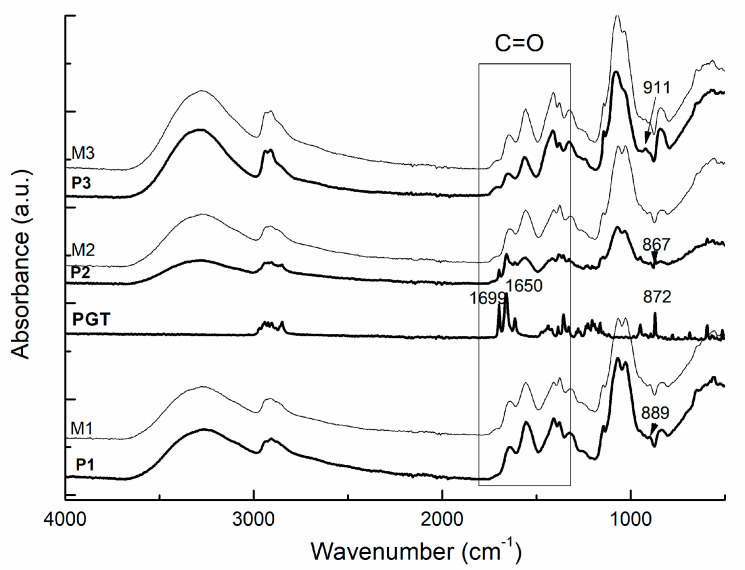
FTİR spectra of the studied formulations and pure progesterone. P1: CS-g-PNIPAAm/PVA *80/20* +PGT; M1: CS-g-PNIPAAm/PVA *80/20*; P2: CS-g-PNIPAAm/PVA *95/5* +PGT; M2: CS-g-PNIPAAm/PVA *95/5*; P3: CS-g-PNIPAAm/PVA *75/25* +PGT; M3: CS-g-PNIPAAm/PVA *75/25*.

The FTIR spectra of chitosan-graft-PNIPAAm/PVA based hydrogels loaded and unloaded with progesterone are presented in Figure 2. From the presented spectra, the characteristics peaks of chitosan, PNIPAAm, and PVA were assigned: particularly forchitosan at 2921–2887 cm^−1^ (–CH_3_, –CH_2_), 1636 cm^−1^ (C=O stretch vibration), 1548 cm^−1^ (secondary amide), 1655 cm^−1^ (amide band I), and 1068–1020 cm^−1^ (C–O stretching of saccharide moiety); PNIPAAm moieties from the graft copolymer were observed at 1454 cm^−1^ (–CH_3_) and 1641 cm^−1^ (O=C). The absorption band around 3310 cm^−1^, becoming wider, indicated hydrogen bonds formation between –OH and –NHCO groups. By varying the amount of PVA, the peaks observed at 3440 and 2921 cm^−1^ due to the –OH and CH_2_ stretching vibrations got wider.

The progesterone’s specific absorption bands were identified by the presence of carbonyl (C=O) stretching band at 1650 cm^−1^. The characteristic band of the double bond of progesterone is generally located between 850 and 900 cm^−1^. For the studied hydrogels, P1, P2, and P3, the progesterone’s specific band from 870 cm^−1^ was determined at 911, 867, and 889 cm^−1^ for P3, P2, and P1, respectively. The shift to lower and/or higher vibration bands indicated the interactions between the polymeric and progesterone’s functional groups, confirming the entrapment of the drug inside the network of the copolymer.

### 2.2. Rheological Characteristics

**Figure 3:**
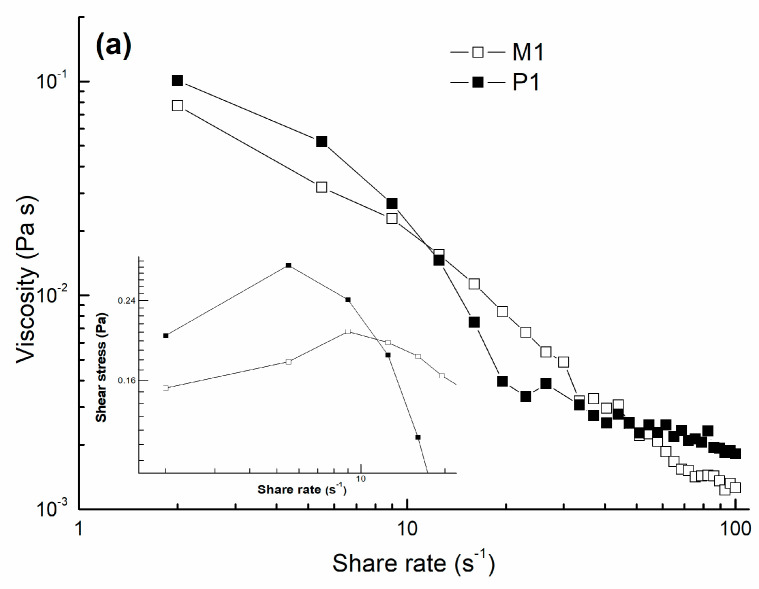

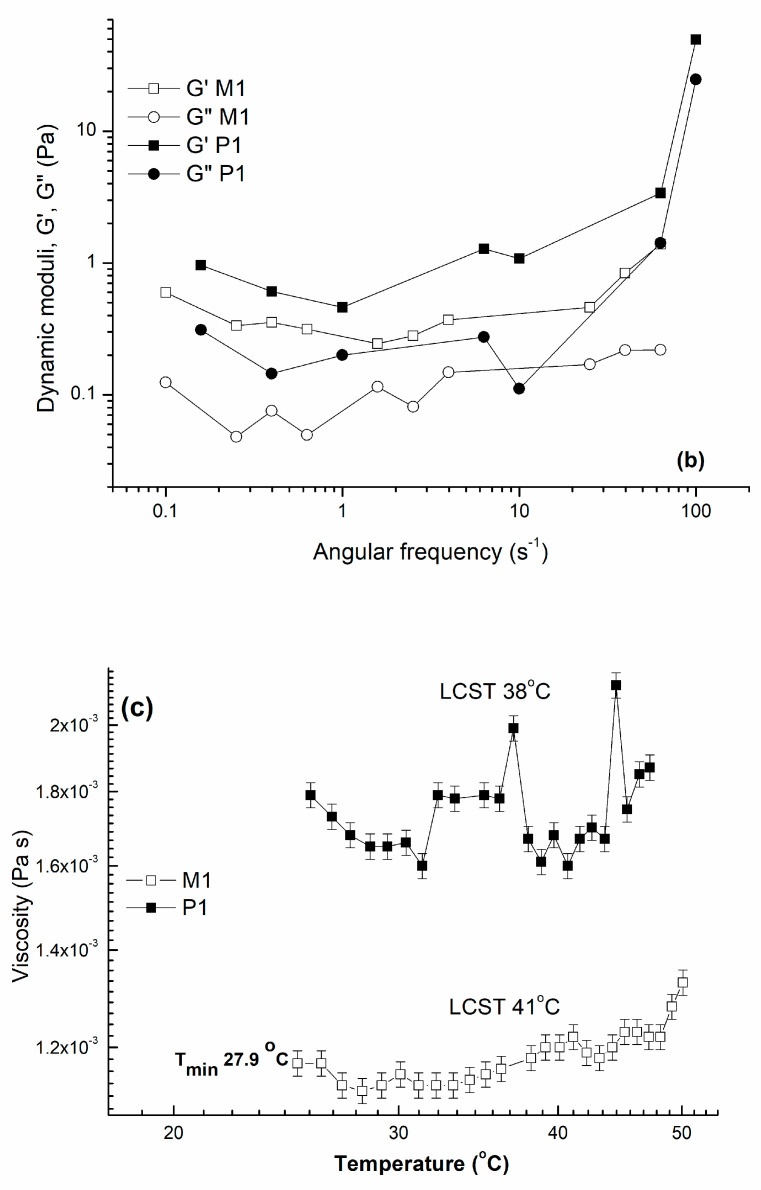
Rheological curves of CS-g-PNIPAAm/PVA 80/20 (M1) and CS-g-PNIPAAm/PVA 80/20-PGT (P1). (**a**) Viscosity dependency on the share rate and share stress–share rate curves; (**b**) Variation of dynamic moduli, storage G’, and loss G’’ moduli with the angular frequency; (**c**) Temperature sweep curves.

The rheological characteristics are shown in Figure 3. 

In Figure 3a, the flow behavior of a selected formulations (M1 and P1) is represented, particularly CS-g-PNIPAAm/PVA 80/20 and CS-g-PNIPAAm/PVA 80/20-PGT.

Figure 3a presented the flow behaviour of solutions obtained from thehydrogels M1 and P1. It was deducted that tat low share rates an initial shear thickening effect followed by shear thinning at higher share rates occurred. The viscosity of the progesterone-loaded formulation (P1) was higher indicating the presence of the drug inside the polymeric network. A key parameter which gives an idea if the formulation is proper for topical application is the dependence of the stress-strain curves (Figure 3a inset); the trendline of the curved presented a pseudo-elastic behavior of the polymeric based solutions which is a favorable characteristic for the purpose of the study. The gel character of the formulations was confirmed by the variation of dynamic moduli (storage G’ and loss G” moduli) at angular frequency modification. Both formulations showed values of G’ higher than G” indicating the gel-alike character; however, the trendline of the curves is not a parallel one, showing a weak gel with tendency to have a crossover point at higher angular frequencies (Figure 3b). For topical application such as mucosal or vaginal when the amplitude of oscillations is low, the values of dynamic moduli at low frequencies are considered. Temperature responsivity was verified with the occasion of the temperature sweep test when the variation of viscosity against temperature rise was determined and shown in Figure 3c. Formulations M1 and P1 started to have a thermo-thickening behavior over lower critical solution temperature (LCST) of PNIPAAm moieties being shifted due to the grafting reaction, crosslinking with PVA, and incorporation of progesterone. Rheological measurements confirmed the previously obtained results applied for mucosal substrate and it showed the reliability for the intravaginal based formulations [19].

### 2.3. Hydrogels Morphology

**Figure 4:**
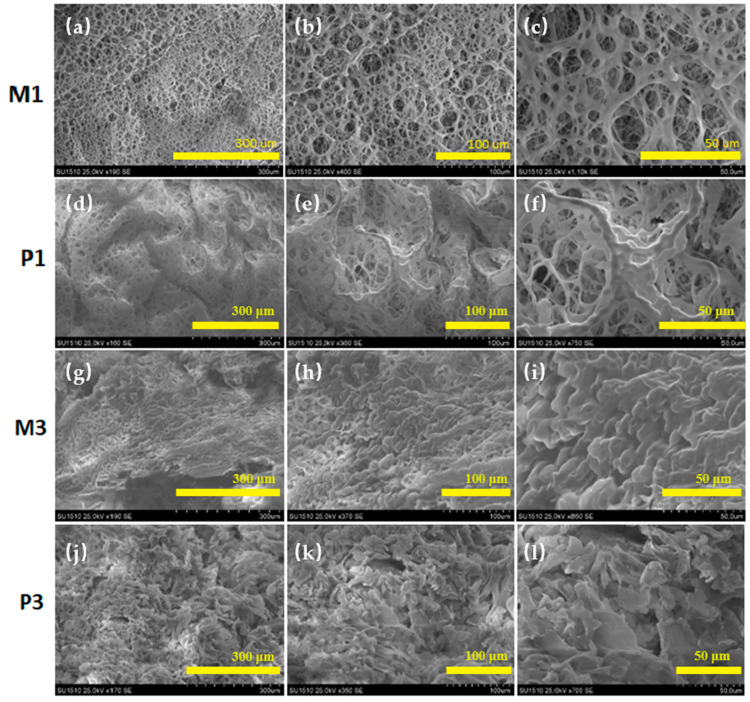
(a–f) SEM images of freeze-dried CS-g-PNIPAAm/PVA 80/20 (M1, P1) and (g–l) 75/25 (M3, P3). P1: CS-g-PNIPAAm/PVA *80/20* +PGT; M1: CS-g-PNIPAAm/PVA *80/20*; P3: CS-g-PNIPAAm/PVA *75/25* +PGT; M3: CS-g-PNIPAAm/PVA *75/25*.

Freeze-dried hydrogels loaded and unloaded with progesterone were analyzed in terms of morphology by SEM microscopy. Figure 4 presents the images obtained at various magnifications to better observe the effect of crosslinking and the addition of drug inside of the network.

As it can be observed within Figure 4a–c,g–i, a porous-like structure was observed for the polymeric matrices without progesterone. At higher magnifications, by comparing Figure 4c,i, it was confirmed that the higher the crosslinking agent, the denser the structure obtained. With the incorporation of progesterone, a less porous morphology was observed (Figure 4d–f for P1 and Figure 4j–l for P3) and the present regular edges were assigned to the progesterone’s molecules confirming the loading within the polymeric matrices According to the obtained SEM images, the distribution of the drug particles was uniform showing a filling effect of the polymeric porous morphology. 

### 2.4. Swelling Ability of CS-g-PNIPAAm/PVA

**Figure 5:**
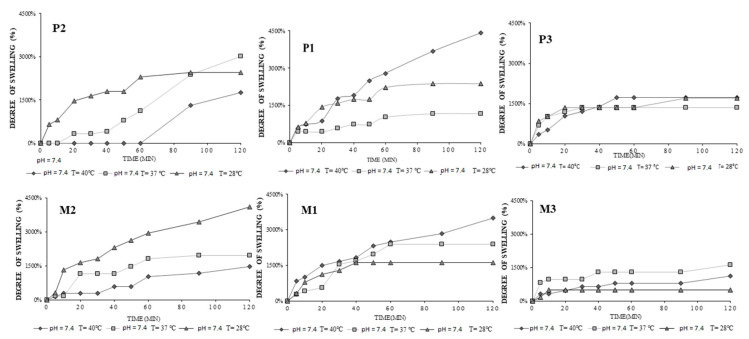
The influence of temperature on the swelling degree, at pH = 7.4. P1: CS-g-PNIPAAm/PVA *80/20* +PGT; M1: CS-g-PNIPAAm/PVA *80/20*; P2: CS-g-PNIPAAm/PVA *95/5* +PGT; M2: CS-g-PNIPAAm/PVA *95/5*; P3: CS-g-PNIPAAm/PVA *75/25* +PGT; M3: CS-g-PNIPAAm/PVA *75/25*.

The swelling ability of the hydrogels loaded (P series) and unloaded (M series) was analyzed and the swelling degrees of the hydrogels were determined. The swelling profile of the hydrogels is represented in Figure 5. 

As shown in Figure 5, all the samples showed degree of swelling ranging from 317% to 4421% up to the pH and temperature of the media.

Previously, the chitosan copolymer was proven to possess temperature and pH responsivity [19,25] similarly with the formulations based on voriconazole; hydrogels containing progesterone exhibited temperature and pH responsivity with respect to the swelling degree (Q%) and release profile. Another factor influencing the swelling and release parameters was the crosslinking degree given by the addition of polyvinyl alcohol (PVA). As it can be observed, Q% had the highest values for sample P1, having least PVA content, particularly at T = 40 °C; determined Q was 4412%, being increased once the temperature was rising. This fact can be explained by the presence of the PNIPAAm segment which induced to the copolymer a LCST temperature of 41 °C [25]. With the increasing of the PVA content, the hydrogels network became denser, and the matrix was not able to absorb water in the same manner, resulting in a lower swelling degree. 

**Figure 6:**
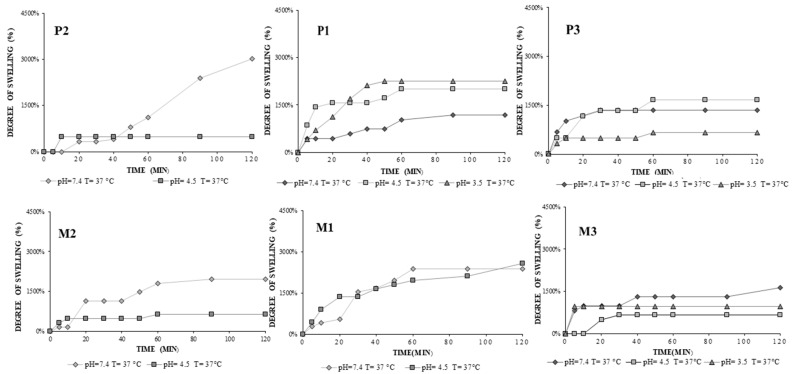
The influence of pH of the media on the swelling degree, at 37 °C. P1: CS-g-PNIPAAm/PVA *80/20* +PGT; M1: CS-g-PNIPAAm/PVA *80/20*; P2: CS-g-PNIPAAm/PVA *95/5* +PGT; M2: CS-g-PNIPAAm/PVA *95/5*; P3: CS-g-PNIPAAm/PVA *75/25* +PGT; M3: CS-g-PNIPAAm/PVA *75/25*.

The observed swelling profiles for the progesterone loaded hydrogels (P1, P2, P3) and progesterone free hydrogels (M1, M2, M3) as function of temperature and pH of the media as following: at pH 7.4, and T = 37 °C, M2 = 1967%, and P2 = 3016%, M1 = 2394%, and P1 = 1176% and M3 = 1639%, and P3 = 1356%. By comparing hydrogels obtained previously with voriconazole [19] and with the progesterone ones, the swelling profiles are slightly different; this may be since progesterone is a more hydrophobic molecule with a high molecular weight, and it can prevent water absorption.Materials which shrink and swell in response to small variations of pH, temperature, or other stimuli affecting the environment are of great interest in many applications, because of their hydrophilicity, biocompatibility, and soft physical properties associated with living tissues, as well as strong control of these in response to external changes which allow for effective treatment [28]. The pH dependence was obviously observed in Figure 6.

The studied hydrogels showed significant Q variations depending on the pH value. At pH 3.5, Q = 2254% for P1, Q = 656% for P3, at pH 4.5, Q = 2000% for P1, Q = 472% for P2, and Q = 1667% for P3, Q did not show significant differences compared to pH 7.4, except for P2, which at pH 7.4, Q reached the value of about 3000%. Moreover, for P2 it was not possible to determine the degree of swelling at pH 3.5, due to the 95% chitosan graft content it dissolved. The 25% cross-linked structure of the P3 hydrogel did not allow a rapid dissolution as in the case of P2. In acidic medium, the swelling ratio had lower values; it was aroused by the screening effect of the counter ions (e.g., OH^−^) which reduce the effective charges of -NH_3_^+^ and therefore decrease the efficient repulsion between the two cations (-NH_3_^+^). However, in a pH near neutral (e.g., pH 7.4), most acid and base groups are nonionized [29]. Bacterial flora showed a lower level of protection and was more prone to bacterial infections. The pH value tends to increase as an inflammatory response to infection. If the pH value exceeds the threshold of 4.5 and tends towards a neutral pH, it results in a favorable environment for the multiplication of microbial factors.

One mechanism that may explain the antimicrobial mode of action results from the ability of polycationic chitosan to interact with the electronegative microbial cell surface which disrupts cell permeability. Positively charged chitosan modifies the bacterial cell surface. The structure of chitosan influences its interaction with mucin in cervicovaginal mucus. Cervicovaginal mucus has a typical acidic pH (3.5–4.5) and the main components of secreted mucus are soluble mucins [20,30].

**Figure 7:**
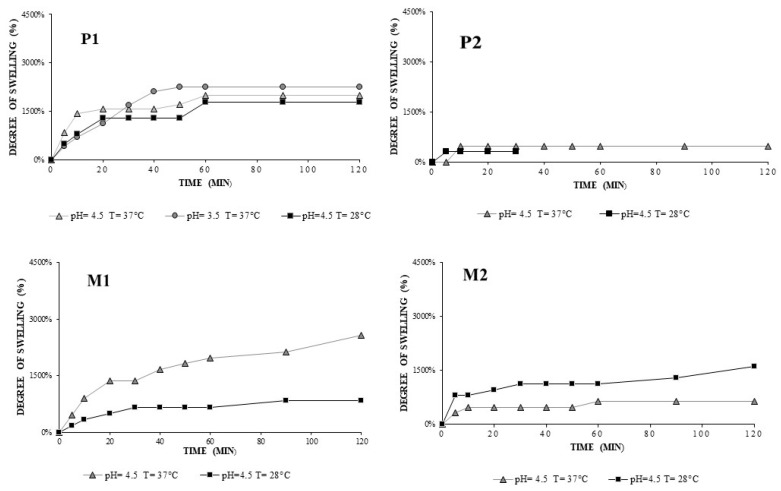
Swelling profiles at pH 4.5 and different temperatures for P1/M1 and P2/M2 hydrogels.

The degree of swelling profiles for hydrogels of P1/M1 and P2/M2 hydrogels at pH 3.5 and 4.5 were represented within Figure 7and it may give an idea on how the hydrogels would swell in the physiological medium The hydrogels resisted 30 minutes without being dissolved in swelling mediumpresenting lower swelling degree values at pH 3.5 and 4.5 compared to the ones obtained at pH 7.4.

**Figure 8:**
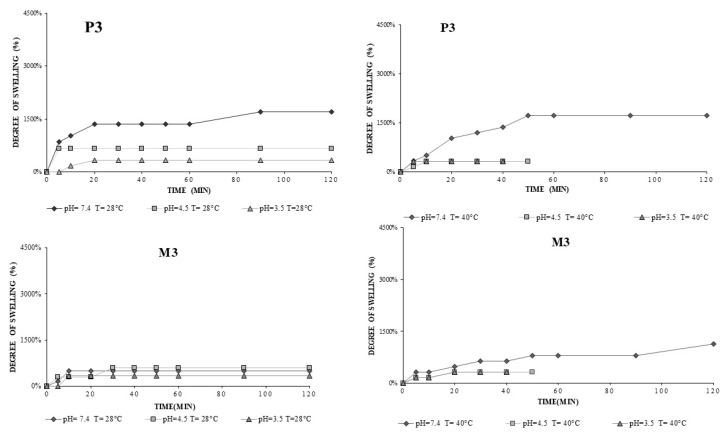
Swelling profiles at temperature T = 28 °C and T = 40 °C and different pH of the media for CS-g-PNIPAAm/PVA *75/25* +PGT (P3) and CS-g-PNIPAAm/PVA *75/25* (M3) hydrogels.

Hydrogel P3 allowed the degree of swelling to be determined at all three pHs in Figure 8. The degree of crosslinking influences the solubility of the hydrogel; P3 is more resistant in the acid environment compared to P1 and P2, because of a larger amount of crosslinking agent.

The degree of swelling of P3 hydrogel (75/25 +PGT) reached the highest value at pH 7.4, T = 40 °C, Q = 1724% (Figure 8); under the same conditions, M3 had Q = 1129% (Figure 8), which indicates that the presence of progesterone can modify the degree of water absorption. At 40 °C the dissolution phenomenon occurred, so at pH 3.5 and 4.5, after the 40 min interval, the hydrogels dissolved; however, P3 having a higher concentration of crosslinker did not dissolve as fast as P2 and P1 (Figure 7). At 28 °C, P3 and M3 reached equilibrium swelling within the first 10 min. The pH value significantly influences Q, which is influenced by deprotonation of chitosan once the pH increases but also by electrostatic repulsion between molecules [29]. PVA is a hydrophilic polymer, but the affinity of the polymer towards its water depends on the degree of hydrolysis as well as the length of the chains. We chose a strongly hydrolyzed PVA, which requires high dissolution temperatures (~100 °C) and a time of about 30 min to break the strong hydrogen bonds formed intra- and intermolecularly. The lower the degree of hydrolysis, the lower the solubility due to the presence of a higher number of hydrophobic groups [22]. 

### 2.5. In Vitro Release of Progesteron

Prior to testing the ability of in vitro delivery of the polymeric matrices, drug loading measurements were performed by using spectrophotometric method UV-VİS. The spectral characteristic peak of progesterone was used; particularly, the absorbance was measured at a wavelength of 270 nm and the quantification of the amount was done by using a calibration curve. The calibration curve was done by plotting the area of the peaks of the standard solutions within the range 0.1–5 mg/mL. The calculation of the unknown concentrations was done by using the calibration curve’s equation (y = 2.1301x − 0.0696, R^2^ − 0.98). According to the results obtained from the loading tests, the total amount of PGT retained within the polymeric matrices are 41% for P1, 58 % for P2, and 36% for P3 with respect to the initial loaded amount (0.5% against polymeric matrix).

**Figure 9:**
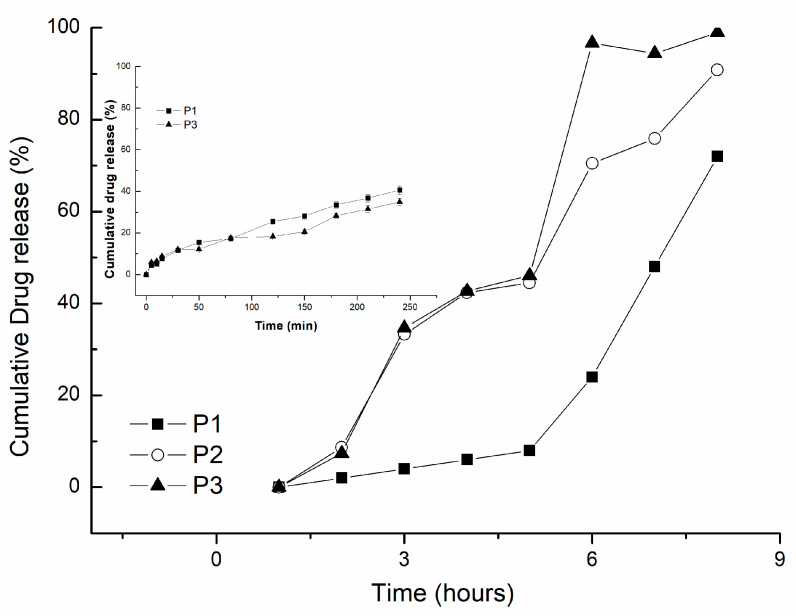
Drug release profiles for CS-g-PNIPAAm/PVA *80/20* +PGT (P1), CS-g-PNIPAAm/PVA *95/5* +PGT (P2), and CS-g-PNIPAAm/ PVA *75/25* +PGT (P3) hydrogels.

In vitro release ability of the studied hydrogels was tested obtaining the release profile.Within Figure 9, the in vitro release profiles of progesterone were presented. 

As presented within Figure 9, an initial burst effect was observed in the first 30 min, being released around 16% from the total amount released. The release profile reached a plateau after 6 h, the total amount release being of 90 % from the total amount loaded. The crosslinking procedure seemed to affect the release profile in the way that the higher the crosslinking degree, the slower the release was. The swelling profile of the hydrogels was correlated with drug release capacity as the determined mechanism being diffusion and swelling controlled ones.

By physically cross-linking with PVA, the density of the polymeric network got higher, with the hydrogels presenting a more stable structure and more difficult to dissolve in acidic media. The concentration of progesterone in P3 is lower compared to P1. In acidic medium, the increase of hydroxyl groups limits the release of the drug due to the interaction of H-bonding with -OH groups [30,31].

The obtained results were supported also by the kinetic parameters calculated based on the release profile and applying Korsmeyer–Peppas Equation (1) summarized within Table 1. The Korsmeyer–Peppas model describes the kinetics of progesterone release at pH 7.4 as shown in Table 2 and Figure 9.

(1) $$F=\frac{M_{t}}{M}=K_{m}\;t^{n},$$

where *F* = fraction of drug release at time “*t*”; *M_t_* = amount of drug released at time “*t*”; *M* = total amount of drug in dosage form; *K_m_* = kinetic constant; *n* = diffusion or release exponent; and *t* = time (h).

The “*n*” value obtained from the slope of the plot calculated based on Korsmeyer–Peppas model indicates the release mechanism of the active compound (Table 1).

In the Korsmeyer–Peppas model, the *n* value was applied to characterize the progesterone release mechanism as described below in Table 2.

The mechanism of drug release from the P1 hydrogel was characterized by the value of the diffusion exponent (*n*). At *n* = 0.562, the diffusion of P1 hydrogel was non-Fickian. Moreover, the amount of progesterone released in the first 60 min was 47.53% for P1 and 28.33% for P3 (1 h). Drug release kinetics was slower for P3, higher crosslinked hydrogel. The value of the diffusion exponent, *n* < 0.5, indicates that we have a quasi-Fickian diffusion (*n* = 0.330); this slow diffusion behaviour may be associated with the degree of cross-linking of the hydrogel. Since P3 is a more cross-linked hydrogel than P1, the weak swelling degree performance results in a slower diffusion time of the progesterone.

The release profile of PGT is closely related to the polymer ratio in the hydrogel content. The increased amount of grafted chitosan in P1 confers quick hydration and relaxation of the polymer chain leading to enhanced drug release [33].

By comparison with a commercial product containing an active substance which has the same purpose to support of the luteal phase, commercial gel dissolved completely after 30 min [34], but the formulations P3 and P1 had a more resistant solubility profile in the vaginal environment, remaining at the action site at least 120 min favorizing the full adsorption of the drug through the mucosa.

As concerning the dosage and to avoid the overdosing causing patient discomfort, the medical recommendations, in the case of in vitro fertilization cycles, for support of the luteal phase, prescribed oral (40 mg/day) and intramuscular (100 mg/day) PGT administration. While the commercial gel containing PGT must be administrated twice a day (one dose containing 90 mg progesterone), the newly prepared hydrogels (P1) showed an optimal therapeutic effect, longer residence time, and would release more than 50% PGT in the first 60 min. Bourgain et al. demonstrated that luteal endometrial maturation is sustained by administrating 300–600 mg PGT, which indicated that P1 hydrogel could contain approximately double the amount of a single dose of crinone gel [35,36,37]. Therefore, the prepared hydrogel showed comparable characteristics with others proposed in the literature and it can be considered that the hydrogel may be loaded to contain a higher or double amount of PGT to deliver the necessary amount of PGT or it can easily plan the exact amount knowing its loading and delivery characteristics.

### 2.6. Bioadhesion Properties

The mucoadhesive strength is closely related to the interaction between the electrostatic attractive force of the polycation with the negatively charged glycoproteins of the mucin, complemented by H-bonding and hydrophobic bonding. The mucin are highly glycosylated glycoproteins consisting of approximately 500 kDa units, which subunify via disulfide bridges to create large water-wrapping structures, with the role of providing a viscoelastic protective layer on the mucosal surface [38].

Chitosan can exert its mucoadhesive capacity at pH < 6. The pH of the environment is a critical factor in mucoadhesion. By its cationic nature, chitosan can prolong its stay time at the mucosal surface due to ionization of the amino groups in the structure.

**Figure 10:**
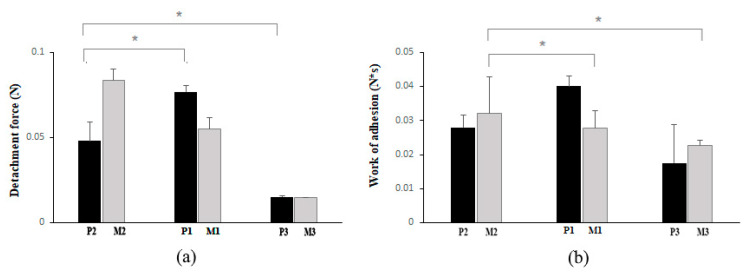
(**a**) Detachment force (N) at pH = 4.5. (**b**) Work of adhesion (N*s) at pH = 4.5. Values were expressed as the mean (SD) from three independent experiments (*n* = 3). Each value represents the mean ± standard error mean (*n* = 3). * *p* < 0.05, One-way ANOVA Turkey HSD post hoc test.

Grafting of chitosan with PNIPAAm allowed chain mobility to control interpenetration between polymer and mucosa, facilitating diffusion, hydrophilicity, and rapid adhesion to the mucosal surface. The bioadhesivity of the hydrogels unloaded and loaded with progesterone was studied by means of the texture analyses. The calculated parameters e.g., the detachment force of the sample from the dialysis membrane surface and the mechanical work of adhesion (product of detachment force and distance), are represented in Figure 10a,b, while the tests aimed to simulate the interaction with the vaginal substrate.

Generally, the connection and formation of interactions between polymer and mucosa involves three steps: a. wetting and swelling of the polymer; b. connection of polymer chains to mucin chains; and c. formation of weak chemical bonds [39,40]. The bioadhesion ratio of hydrogels in acidic media showed promising results; P1 shows higher adhesion properties compared to P2 and P3 due to the higher content of polymeric matrix and thus increased number of functional groups which can adhere to the substrate. The presence of drug within the polymeric matrix seemed to have had effect on the adhesion parameters. By balancing the amount of crosslinking together with the drugs’ molecules, it may be obtained a synergic contribution to higher mechanical parameters. Too high amount of crosslinking agent would make a matrix denser (M3), with drug (P3) ununiformly distributed resulting in a formulation with lower adhesion to the substrate. M2 and P2 by comparison had higher polymeric amount and a degree of crosslinking weaker; the polymeric matrix without drug M2 had a good adherence while the matrix with progesterone (P2) showed lower values with the respect to the detachment forces; progesterone seemed to hinder the matrix characteristics. For the propose of reducing the uterine contractility and to support the luteal phase for in vitro fertilization, higher values of the detachment forces and the work of adhesions are needed and the material P1 would be a good candidate for application.

## 3. Conclusions

Hydrogels are a category of smart biomaterials with remarkable therapeutic applications. Hydrogels based on chemically modified chitosan (chitosan grafted from poly(Nisopropylacrylamide) CS-g-PNIPAAM) were prepared with polyvinyl alcohol (PVA) with various compositions with respect to the chitosan moieties and PVA content. Based on the previous experience with these materials, three compositions were selected with CS-g_PNIPAm 95 wt % (M2), 80 wt % (M1), and 75 wt % (M3). The crosslinking technique with PVA seemed a smart strategy to adjust some of the weak properties of the polymeric matrix such as gel strength and texture. The newly prepared materials were destined to vaginal applications, more specifically to formulations to deliver progesterone which is necessary to support the luteal phase of the pregnancy and to avoid miscarriages.

The interaction between modified chitosan and polyvinyl alcohol (PVA) results hydrogel that can simulate a material which withstand shear forces encountered in the vagina due to its mechanism of swelling once in contact with vaginal fluids. Hydrogels are sensitive to both pH and temperature variations.

The use of polyvinyl alcohol in conjunction with derived chitosan was also aimed to increase the degree of adhesion by interpenetrating hydroxyl groups with amino groups. By balancing some parameters such as degree of crosslinking and the amount of polymeric matrix, an optimal formulation was established as M1 and P1, the progesterone loaded P1.

The thermo-responsive character of the formulations was shown and determined at a temperature close to the body temperature particularly at T = 40 °C. The increased PVA concentration within the cryogel had a negative effect on the swelling and bioadhesion of the hydrogel. The release profile of PGT is closely related to the polymer ratio in the hydrogel content. The increased amount of grafted chitosan in P1 confers rapid hydration and relaxation of the polymer chain leading to an increased release of the drug compared to P3.

Progesterone-loaded hydrogels had more prominent sensitivity than those without the drug, showing the synergic effect of the drug with the polymeric carrier. Taking into account also other studies performed for the same purpose, the obtained results were comparable. The prepared hydrogels showed comparable characteristics with others proposed in the literature and it can be considered that the hydrogel may be loaded to contain a higher or double amount of PGT to deliver the necessary amount of PGT and it can easily plan the exact amount knowing its loading and delivery characteristics; these outcomes may explain the proposal as candidate of the P1 hydrogel (CS-g-PNIPAAm/PVA 80/20 wt%) as vaginal vehicle for progesterone administration.

## 4. Materials and Methods

### 4.1. Materials

Chitosan-graft–poly(N-isopropylacrylamide) copolymer (CS-g-PNIPAAm) [25] was synthesized. The synthesis’ details and experimental set-up were previously presented. The synthesized copolymer was further used for the preparation of cryogels. Polyvinyl alcohol (PVA), 99% hydrolyzed with Mw of 89,000–98,000 Da was achieved from Sigma-Aldrich, St. Louis, MO, USA. Phosphate buffered solution pH 7.4 was prepared with monosodium phosphate NaH_2_PO_4_·2H_2_O, Sigma-Aldrich, Darmstadt, Germany, and disodium phosphate Na_2_HPO_4_, Sigma-Aldrich, Darmstadt, Germany. Phosphate buffered solution pH 4.5 and pH 3.5; citric acid, Sigma-Aldrich, Germany; NaOH, Sigma-Aldrich, Germany, HCl, Sigma-Aldrich, Germany. Progesterone was received from Antibiotice SA Iasi, Romania.

Preparation of Freeze–Thaw Hydrogels CS-g-PNIPAAm/PVA

For the hydrogel’s preparation, 1 wt.% solutions of CS-g-PNIPAAm and 5 wt.% PVA in a ratio of 75/25 *v/v*%, 80/20 *v/v*%, and 95/5 *v/v*% were mixed. The obtained solutions were frozen at −20 °C for 20 h and thawed at 25 °C for 4 h during three cycles. Similar hydrogels with slight modifications with respect to the composition were prepared for prolonged delivery of voriconazole for oral candidiasis [19].

Loading of progesterone was done by mixing the graft copolymer solution with progesterone 0.5 wt.% against the polymeric matrix in an aqueous solution of acetic acid 0.5 wt.%. A solution of PVA, 5 wt.%, was added and freeze–thawing cycles were performed as mentioned above. The final gels were lyophilized to obtain sponge-like materials which were used further for characterization and tests. Table 3 shows the list of studied systems and their codes.

### 4.2. Characterization Methods

Hydrogels based on CS-g-PNIPAAm with various amounts of PVA loaded and unloaded with progesterone were characterized in terms of structural identity and morphology, swelling ability, rheological characteristics, drug delivery capability, and bio-adhesivity onto a substrate.

#### 4.2.1. FTIR Spectroscopy

The composition of hydrogels was investigated by using FTIR. All the spectra were scanned from 4000 to 500 cm^−1^ with a resolution of 2 cm^−1^ by a FTIR spectrometer Platinum—ATR, Ettlingen, Germany. The analyses were performed on freeze-dried samples.

#### 4.2.2. Rheological Measurements

The rheological investigations were performed by using an Anton Paar MCR301 Rheometer, Berlin, Germany equipped with a cone-plate geometry measuring system with a cone angle of 1° and a diameter of 50 mm at different shear rates and angular frequencies. The samples were placed onto the plate for 5 min to eliminate residual shear history, and then experiments were carried out immediately. The measuring device was equipped with a temperature unit that gave good temperature control (±0.05 °C). The rheological tests were done as follows: 

Low behavior (Newtonian or non-Newtonian): The viscosity (η) of the samples were measured as a function of increasing shear rate (γ = 0.1–100 s^−1^). Viscoelastic behavior: Dynamic modulus, storage modulus (G’), and loss modulus (G’’) of the hydrogels were measured as a function of angular frequency (ω = 0.1–100 s^−1^) using oscillatory tests.

Temperature-dependent behavior of hydrogels: The test was performed maintaining both frequency and amplitude constant, the only variable parameter being temperature. The temperature was varied within 25–50 °C and the storage modulus values were plotted against temperature. The transition temperature LCST of the gels was determined.

#### 4.2.3. Scanning Electron Microscopy

Scanning electron microscopy (SEM) was used for scaffolds morphology investigation and images were recorded on a Hitachi SU 1510 Scanning Electron Microscope (Tokyo, Japan). Scaffolds with maximum and minimum content of PVA, loaded and unloaded with Progesterone, were deposited on an aluminum stub and coated with a 7 nm thick gold layer. A Cressington108 device was used for deposition. Finally, the SEM images were visualized, and the data registration was performed at an accelerating voltage of 25 kV.

#### 4.2.4. Swelling Properties

The determination of the degree of swelling was performed by the volumetric method. Using this method, we analyzed the interaction between materials obtained by physical crosslinking and fluids simulating the vaginal environment. A QIAquickRSpin Column 50 device (Miami, FL USA), equipped with a cellulosic membrane that does not allow passage of the sample and interconnected to a microsyringe, was used.

The sample was inserted into the QIAquickRSpin Column 50 device, the cellulosic membrane being pre-wetted with phosphate buffer saline to simulate environmental conditions. A volume of PBS solution (1 mL) was added to the microsyringe which immersed the sample (*m* ≅ 3 mg) to determine the swelling capacity of the hydrogel. The volume absorbed by the probe was determined for 120 min. The degree of swelling (Q) was calculated using the following formulae:

(2) $$Q\;\left(\%\right)=\frac{\left(W_{t}-W_{d}\right)}{W_{d}}\times 100$$

where $W_{t}=W_{d}+V_{abs}\left(t\right)$, *W_t_* is the mass of solution absorbed at time *t*, and *W_d_* is the mass of the sample.

The tests were performed using phosphate buffer solutions (PBS) at different pH: pH 7.4, pH 4.5, and pH 3.5. Samples were placed in the oven at different temperatures: T = 28 °C, T = 37 °C, and T = 40 °C.

#### 4.2.5. In Vitro Release Profiles

The release profile of the model drug (progesterone, PGT) from the prepared hydrogels were evaluated by diffusion experiments using a dialysis membrane, which was previously boiled for 1 h in distilled water at 100 °C. After cooling, the weighed sample (*m* ≅ 30 mg) was added to each dialysis membrane together with 5 mL of buffer solution at pH 7.4.

The dialysis membrane together with the working sample was placed in the centrifuge tube, over which a volume of 30 mL of buffer solution at pH 7.4 was added. All samples were placed in the oven at 37 °C.

The data measurement was done by using a spectrophotometer PharmaSPec UV-1700 (Canton, MA, USA), at a wavelength of 270 nm for 4 h. The release pharmacokinetic parameters (k-release rate) and *n* (release coefficient) were calculated by applying the theoretical approach of Korsmeyer–Peppas.

#### 4.2.6. Bioadhesion Tests

The texture and the mucoadhesion related properties were studied by using a TA.XTplus texture analyser from StableMicro Systems, Surrey, UK. Samples tested with a thickness of 1 mm were attached to a cylinder probe of a diameter of 10 mm. The tissue model used was dialysis membrane, boiled and cooled beforehand, and PBS at pH 4.5, T = 37 °C, V = 20 μL was used to simulate the biological fluid environment.

Test parameters: pre-test speed was 0.5mm/s; test speed: 0.1 mm/s; post-test speed: 0.1 mm/s; applied force 0.5 g; return distance: 15 mm; contact time: 60 s; tare mode: auto; data acquisition rate: 500 pps. By using the Exponent software, the detachment force of the sample from the dialysis membrane surface and the mechanical work of adhesion (product of detachment force and distance) were calculated.

## Figures and Tables

**Table 1:** The release’s kinetic parameters by applying the *Korsmeyer–Peppas* model.

Korsmeyer–Peppas									
Data	*K*			*n*			R^2^		
	1 h	2–4 h	4 h	1 h	2–4 h	4 h	1 h	2–4 h	4 h
P1	0.010	0.026	0.008	0.469	0.318	0.562	0.989	0.997	0.995
P3	0.012	0.031	0.017	0.345	0.214	0.330	0.985	0.929	0.955

**Table 2:** Release mechanism with variation of “*n*” values [32].

Release Exponent	Drug Transport Diffusion	Rate as a Function of Time
*n* < 0.5 (0.45)	quasi Fickian diffusion	*t* ^−0.5^
*n* = 0.5 (0.45)	Fickian diffusion	*t* ^−0.5^
0.5 < *n* < 1 (0.89)	non-Fickian diffusion	*t* ^*n*−1^
*n* = 1 (0.89)	case II transport	zero order release
*n* > 1 (0.89)	super case II trasnsport	*t* ^*n*−1^

**Table 3:** List of hydrogel samples used for the study.

CODE	HYDROGEL	
**P1**	CS-g-PNIPAAm/PVA 80/20 with progesterone	Freeze-dried CS-g-PNIPAAm crosslinked with PVA within a ratio of 80/20 *v/v*%; and loaded with Progesterone (PGT) 0.5%.
**M1**	CS-g-PNIPAAm/PVA 80/20	Freeze-dried CS-g-PNIPAAm crosslinked with PVA ratio 80/20 *v/v*%.
**P2**	CS-g-PNIPAAm/PVA 95/5 with progesterone	Freeze-dried CS-g-PNIPAAm crosslinked with PVA, ratio 95/5 *v/v*% and loaded with PGT 0.5%
**M2**	CS-g-PNIPAAm/PVA 95/5	Freeze-dried CS-g-PNIPAAm crosslinked with PVA, ratio 95/5 *v/v*%.
**P3**	CS-g-PNIPAAm/PVA 75/25 with progesterone	Freeze-dried CS-g-PNIPAAm crosslinked with PVA ratio 75/25 *v/v*% and loaded with PGT 0.5%
**M3**	CS-g-PNIPAAm/PVA 75/25 with progesterone	Freeze-dried CS-g-PNIPAAm crosslinked with PVA ratio 75/25 *v/v*%.
